# Supplementary figures and images for: Uncovering the potential functions of lymph node metastasis-associated aberrant methylation differentially expressed genes and their association with the immune infiltration and prognosis in bladder urothelial carcinoma
Source: PeerJ. 2023 Apr 24;11:e15284. doi: 10.7717/peerj.15284 (PMC10135411; doi:10.7717/peerj.15284)

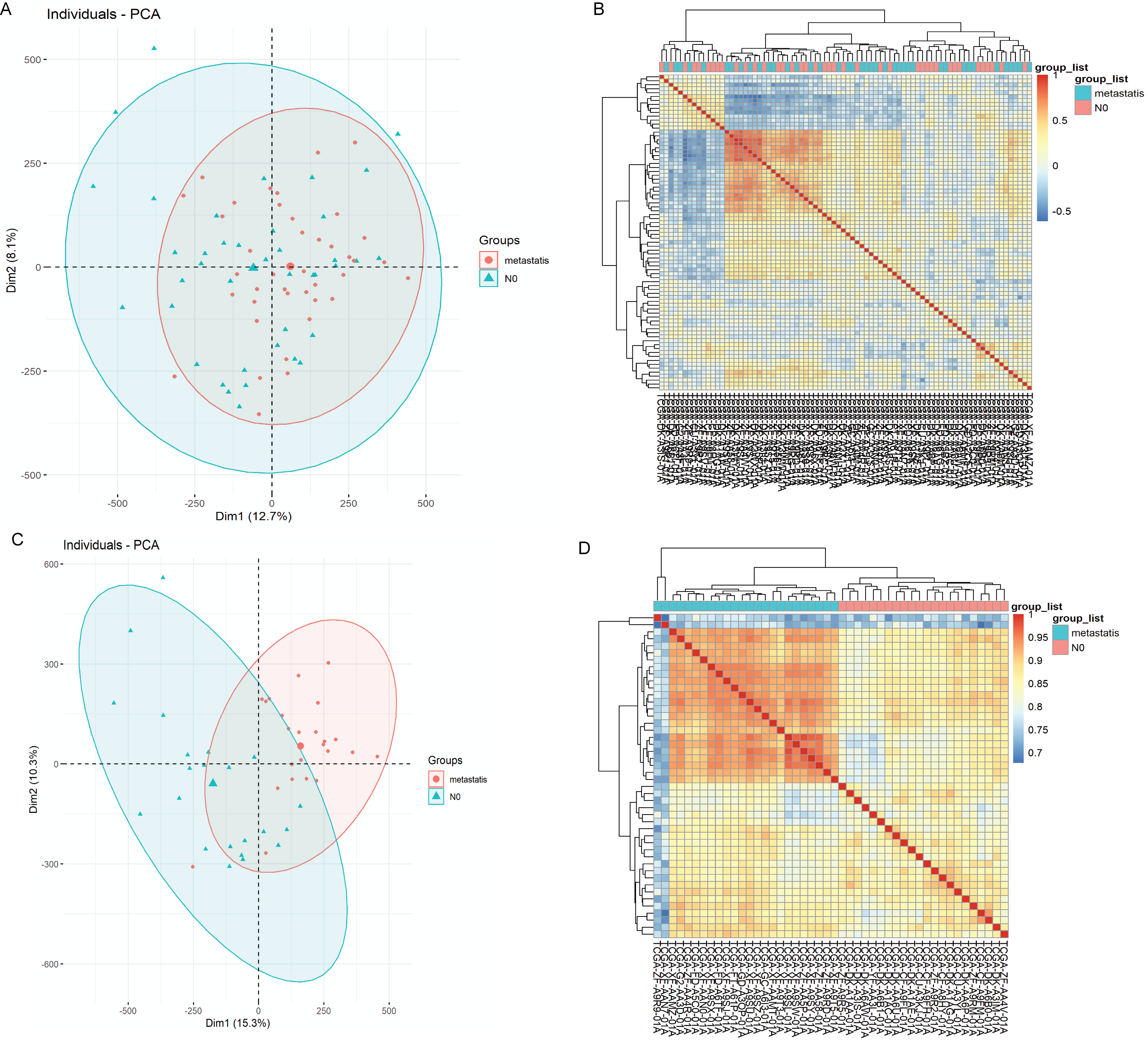

Supplement: Supplemental Information 2 — A: Principal components analysis (PCA) diagram of 40 N0 methylation samples and 40 N1-N3 methylation samples before quality control; B: Heatmap of 40 N0 methylation samples and 40 N1-N3 methylation samples before quality control; C: PCA diagram of 22 N0 methylation samples and 24 N1-N3 methylation samples after quality control; D: Heatmap of 22 N0 methylation samples and 24 N1-N3 methylation samples after quality control. [file peerj-11-15284-s002.png]

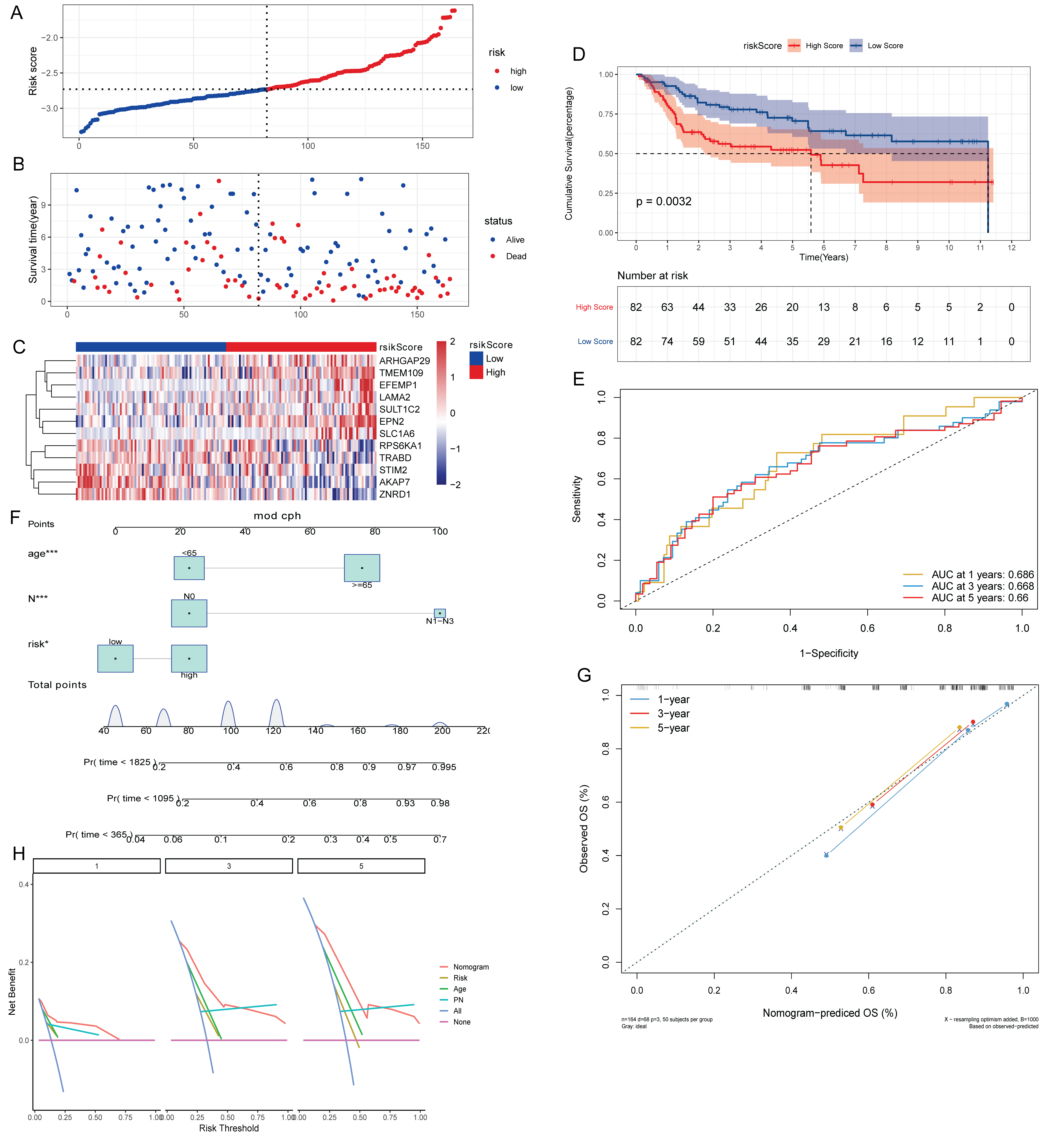

Supplement: Supplemental Information 3 — A: Distribution map of risk score in the GSE13507 dataset; B: Survival status map of patients in high and low risk groups; C: Expression heatmap of 12 signature genes in high and low risk groups; D: The Kaplan-Meier method was used to analyze the survival of patients in high and low risk groups; E: Time-dependent ROC curves were used to validate the prognostic accuracy of the risk score model; F: Nomogram of age, N stage and risk score; G: Calibration curves for nomogram predicting 1-, 3-, and 5-year OS; H: DCA curves of nomogram, age, N stage and risk score at 1-, 3- and 5-years. [file peerj-11-15284-s003.png]

Normal


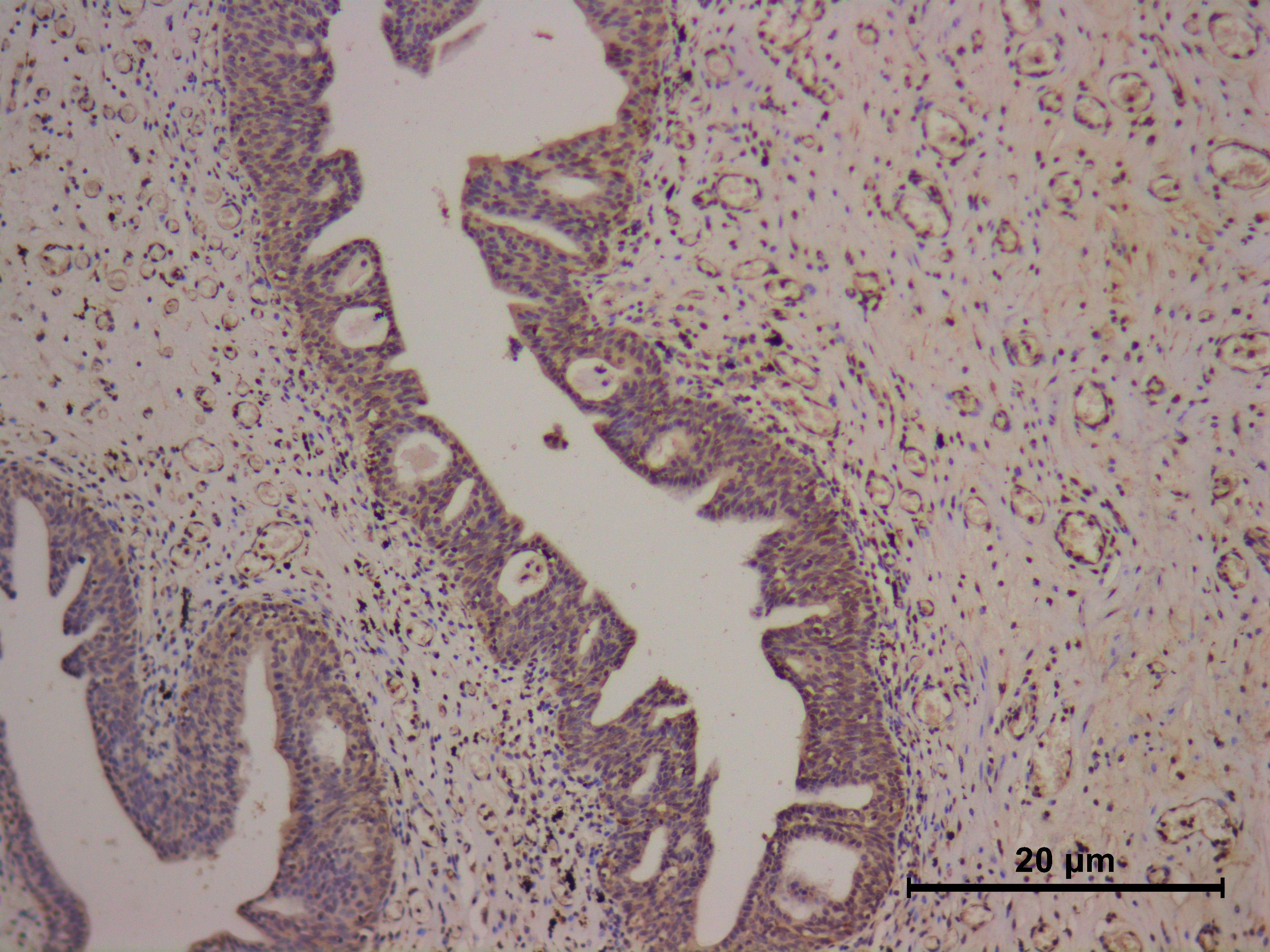


Cancer


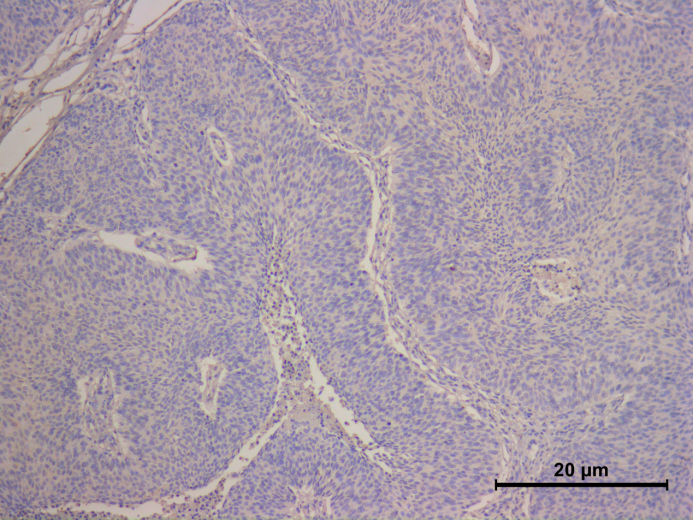

Supplement: Supplemental Information 8 [file peerj-11-15284-s008.docx]

T1


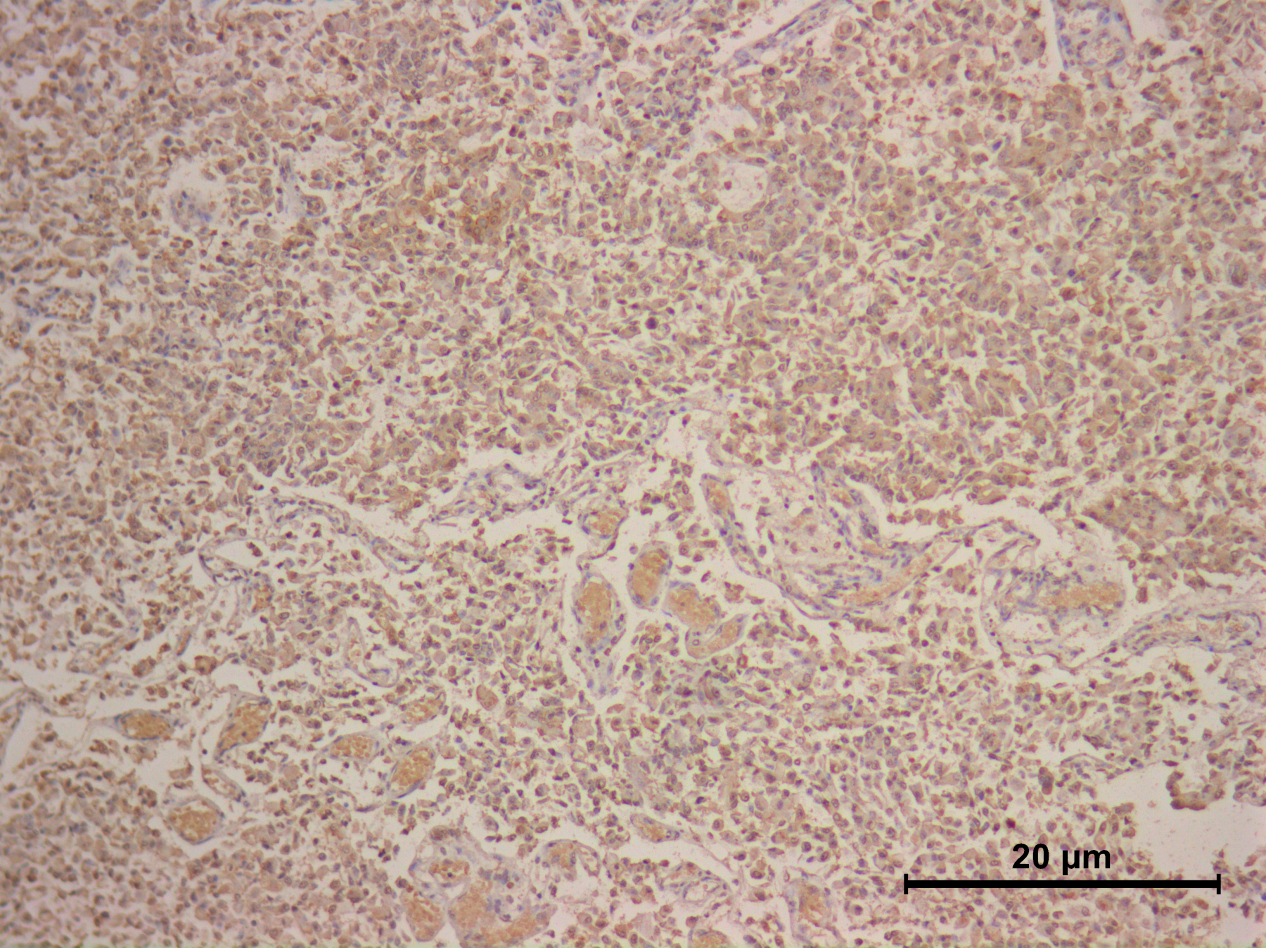


T2


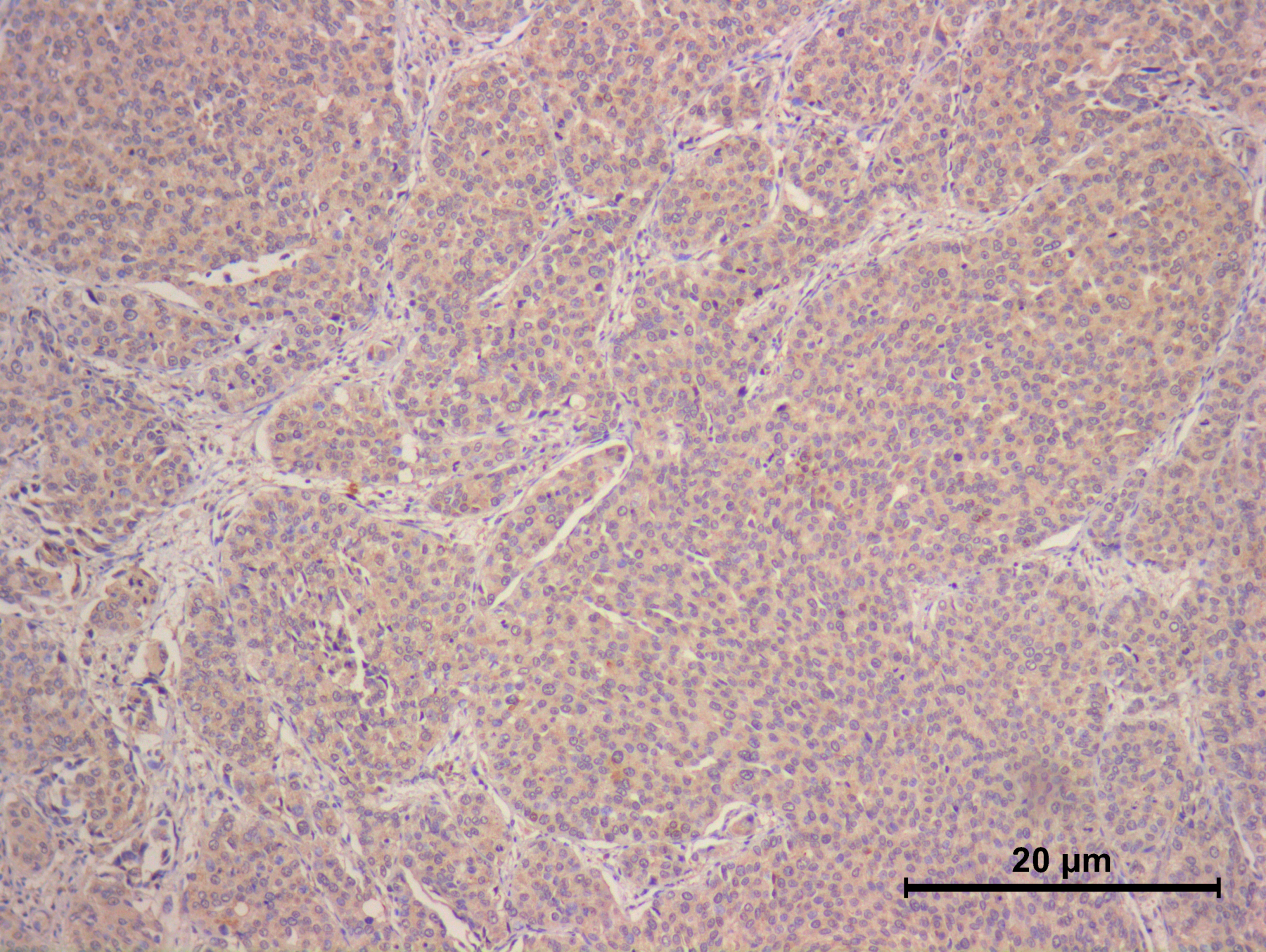


T3


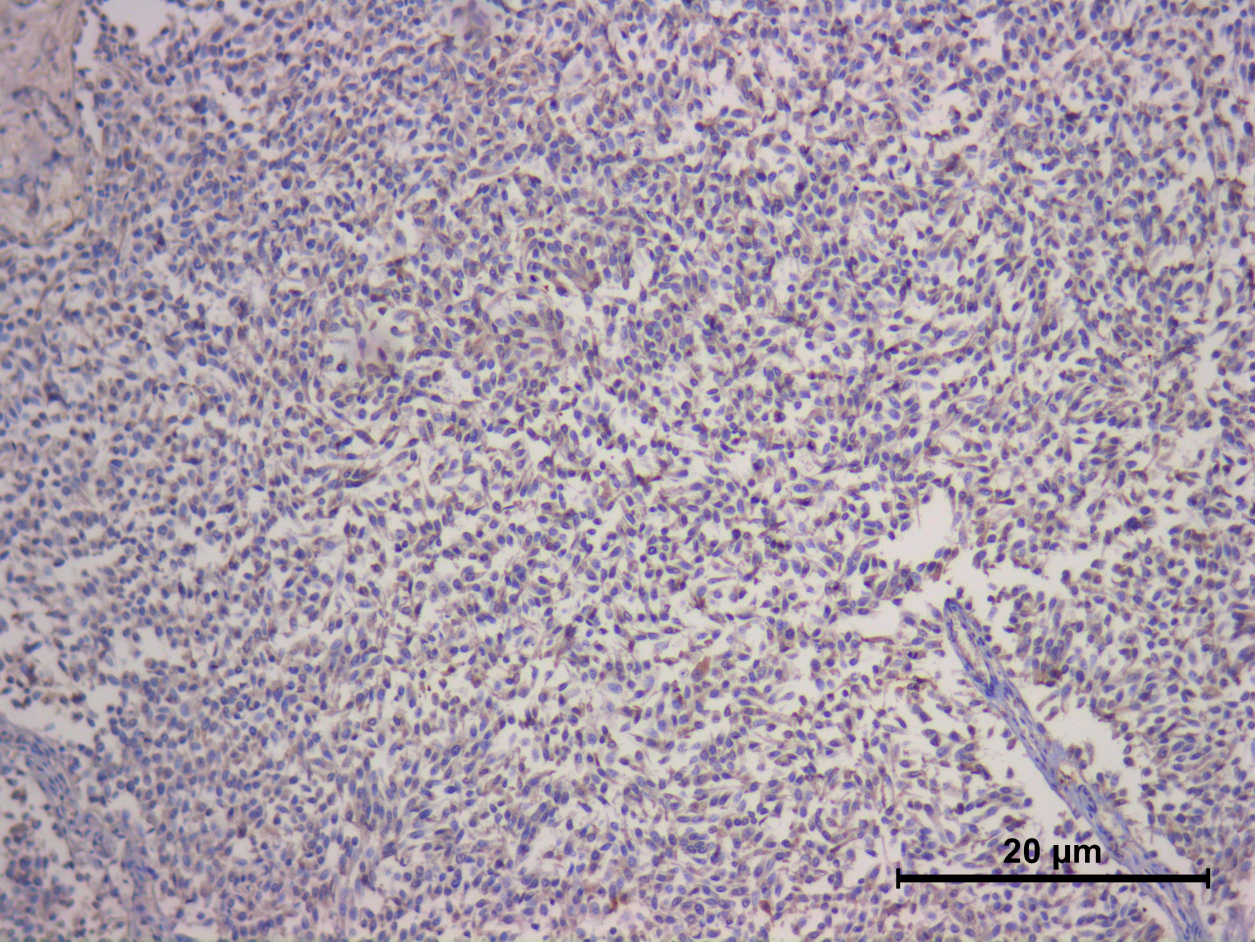


T4


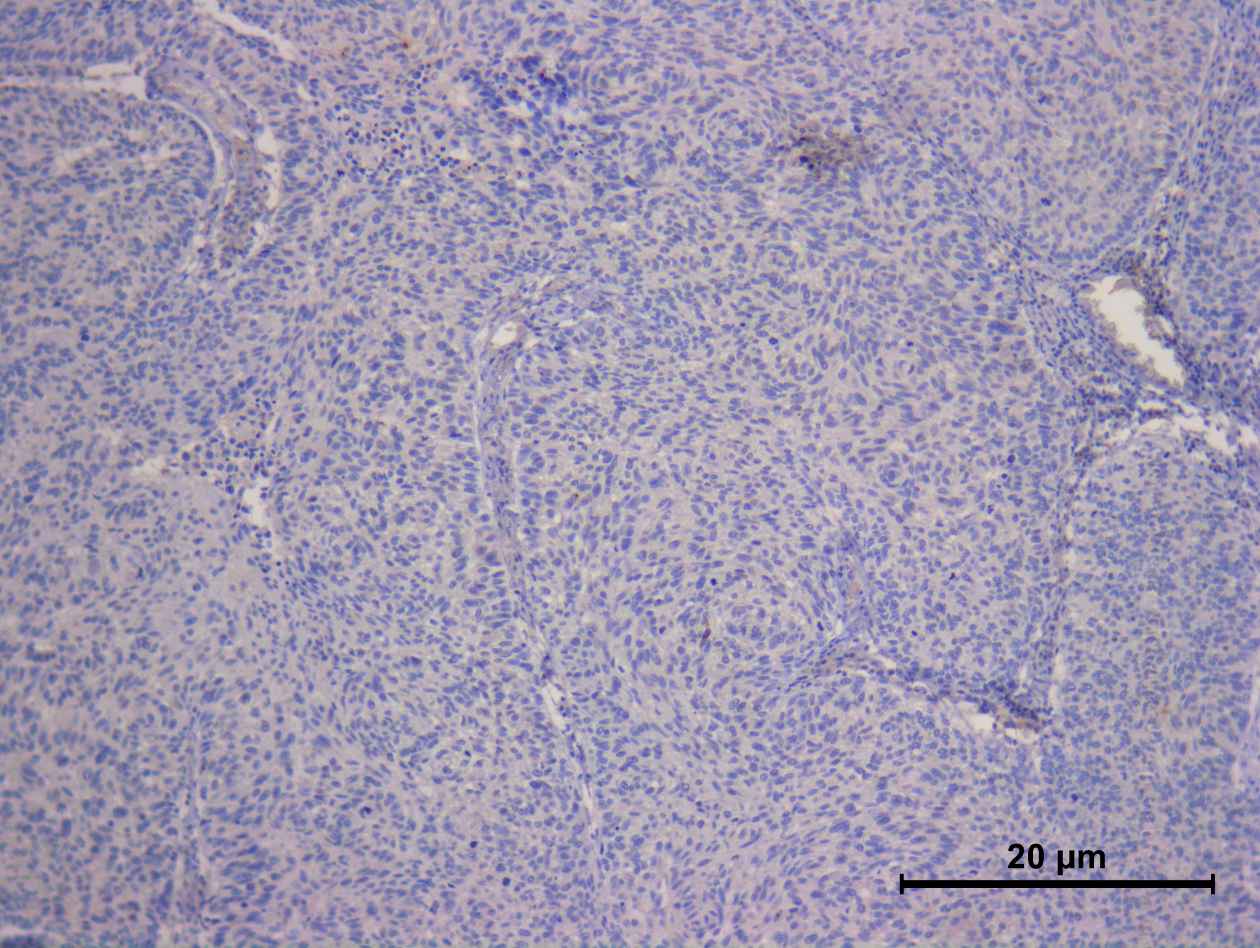

Supplement: Supplemental Information 9 [file peerj-11-15284-s009.docx]

Low grade


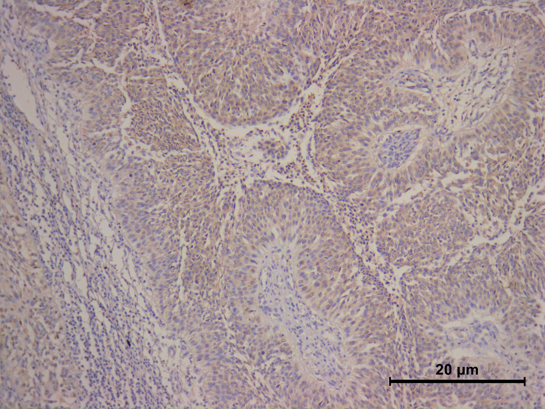


High grade


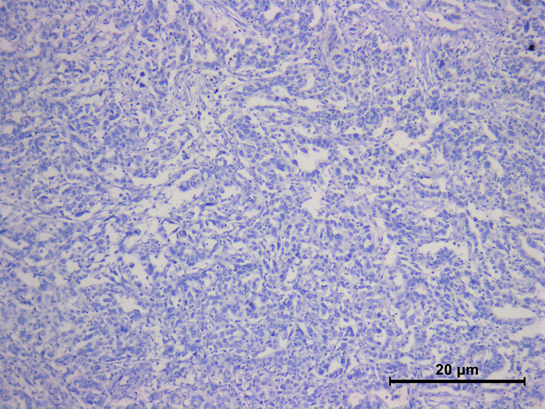

Supplement: Supplemental Information 10 [file peerj-11-15284-s010.docx]

No metastasis


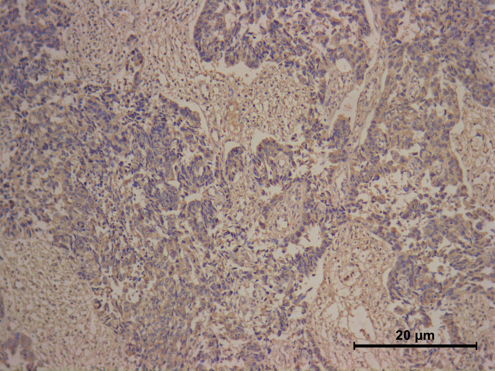


Metastisis


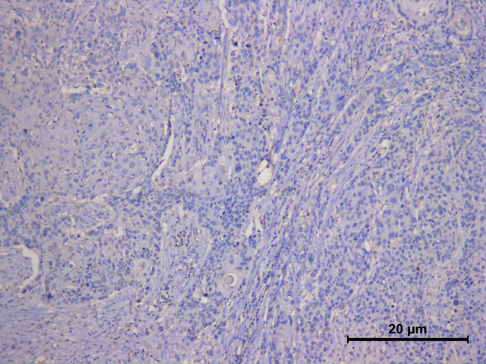

Supplement: Supplemental Information 11 [file peerj-11-15284-s011.docx]
