# Supplementary material for: Uncovering the potential functions of lymph node metastasis-associated aberrant methylation differentially expressed genes and their association with the immune infiltration and prognosis in bladder urothelial carcinoma
Source: PeerJ. 2023 Apr 24;11:e15284. doi: 10.7717/peerj.15284 (PMC10135411; doi:10.7717/peerj.15284)
Supplement: Supplemental Information 4 [file peerj-11-15284-s004.docx]

**Table S1 Sample size and clinical characteristics in TCGA-BLCA and GSE13507 datasets**

| TCGA-BLCA | Type | Risk score | | M stage | | | N stage | | T stage | | | Age | | Gende | | Stage | | |  |
| --- | --- | --- | --- | --- | --- | --- | --- | --- | --- | --- | --- | --- | --- | --- | --- | --- | --- | --- | --- |
|  |  | low | high | M0 | M1 | unknown | N0 | N1-N3 | T1-T2 | T3-T4 | unknown | >=65 | <65 | male | female | stage i-ii | stage iii-iv | unknown |  |
|  | Number | 182 | 181 | 178 | 8 | 177 | 236 | 127 | 104 | 241 | 18 | 228 | 135 | 268 | 95 | 101 | 260 | 2 |  |
| GSE13507 | Type | Risk score | | N stage | | Grade | | Therapy | | | Chemo | | Age | | Gende | | Recurrence | | |
|  |  | low | high | N0 | N1-N3 | low | high | Yes | No | unknown | Yes | No | >=65 | <65 | male | female | Yes | No | unknown |
|  | Number | 82 | 82 | 149 | 15 | 105 | 59 | 56 | 47 | 61 | 26 | 138 | 95 | 69 | 134 | 30 | 36 | 67 | 61 |
