# Supplementary material for: Uncovering the potential functions of lymph node metastasis-associated aberrant methylation differentially expressed genes and their association with the immune infiltration and prognosis in bladder urothelial carcinoma
Source: PeerJ. 2023 Apr 24;11:e15284. doi: 10.7717/peerj.15284 (PMC10135411; doi:10.7717/peerj.15284)
Supplement: Supplemental Information 5 [file peerj-11-15284-s005.docx]

Sv-huc-1 T24 EJ 5637 BIU87


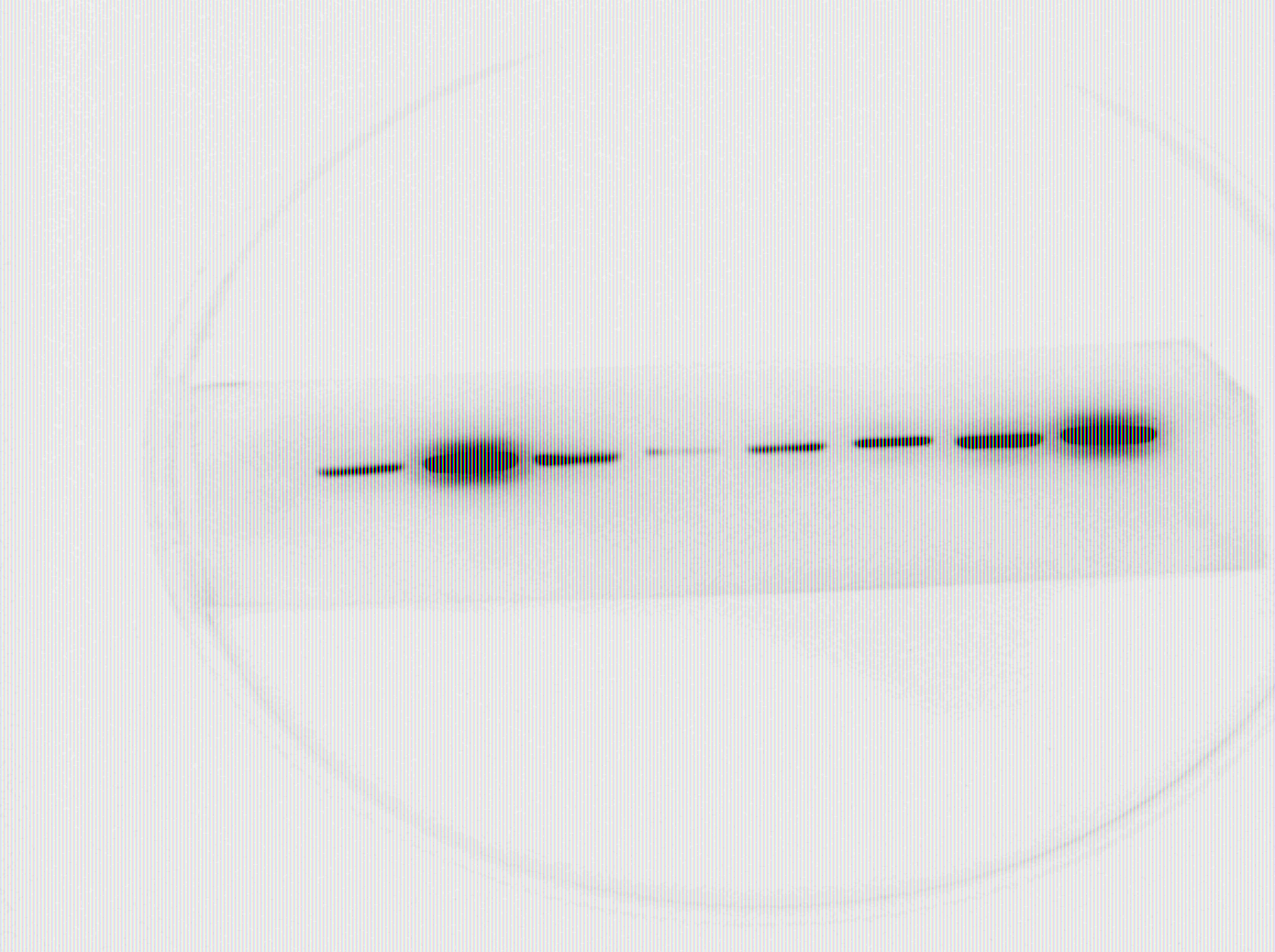


Western blot detection of AKAP7 protein expression level in Sv-huc-1, T24, EJ, 5637, BIU87 cells

Sv-huc-1 T24 EJ 5637 BIU87


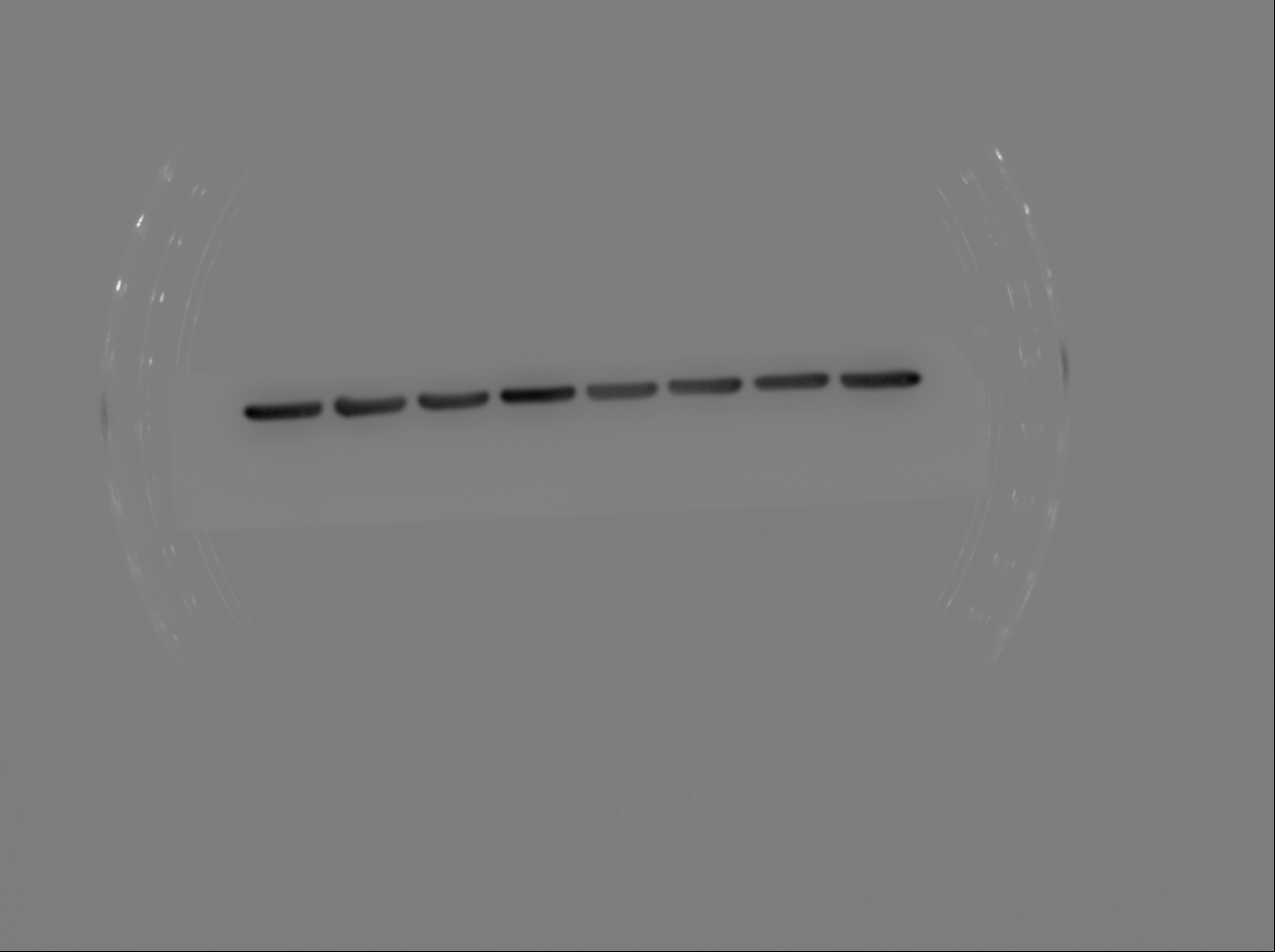


Western blot detection of GAPDH protein expression level in Sv-huc-1, T24, EJ, 5637, BIU87 cells

NC OE1 OE2 OE3


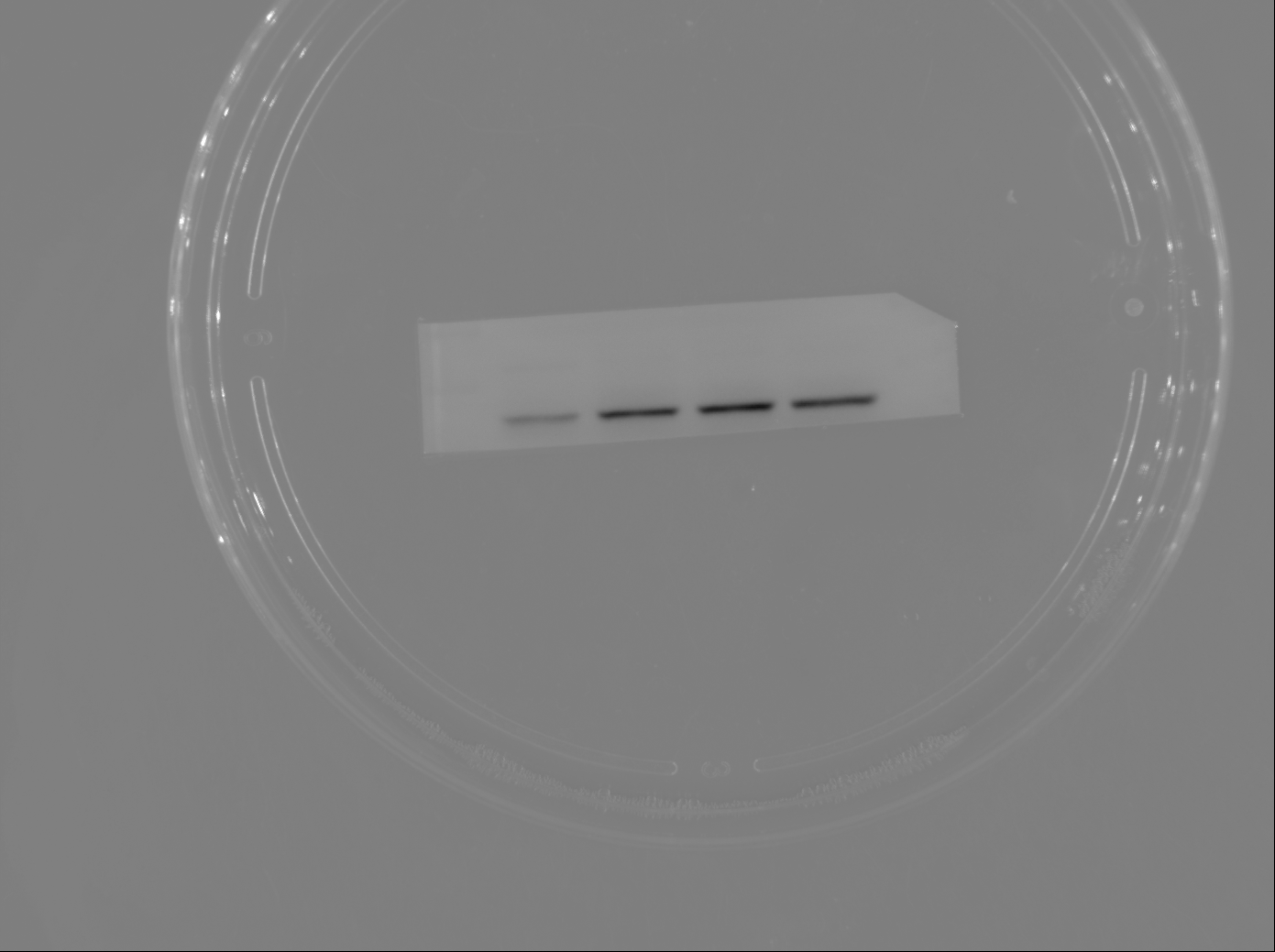


The expression level of AKAP7 protein in EJ cells was detected by WB after overexpression

NC OE1 OE2 OE3


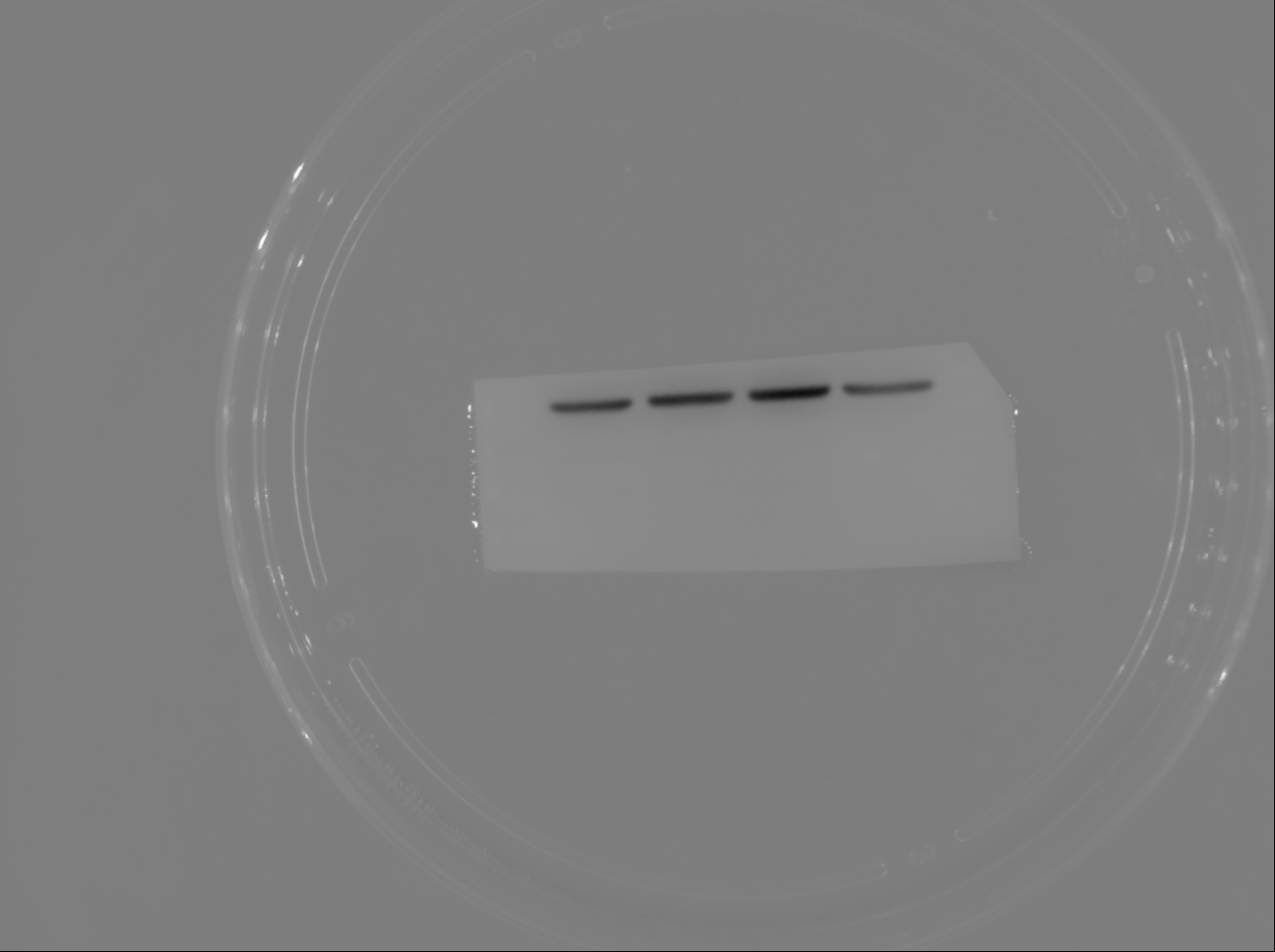


The expression level of GAPDH protein in EJ cells was detected by WB after overexpression

T24NC sh1 sh2 sh3 NC sh1 sh2 sh3


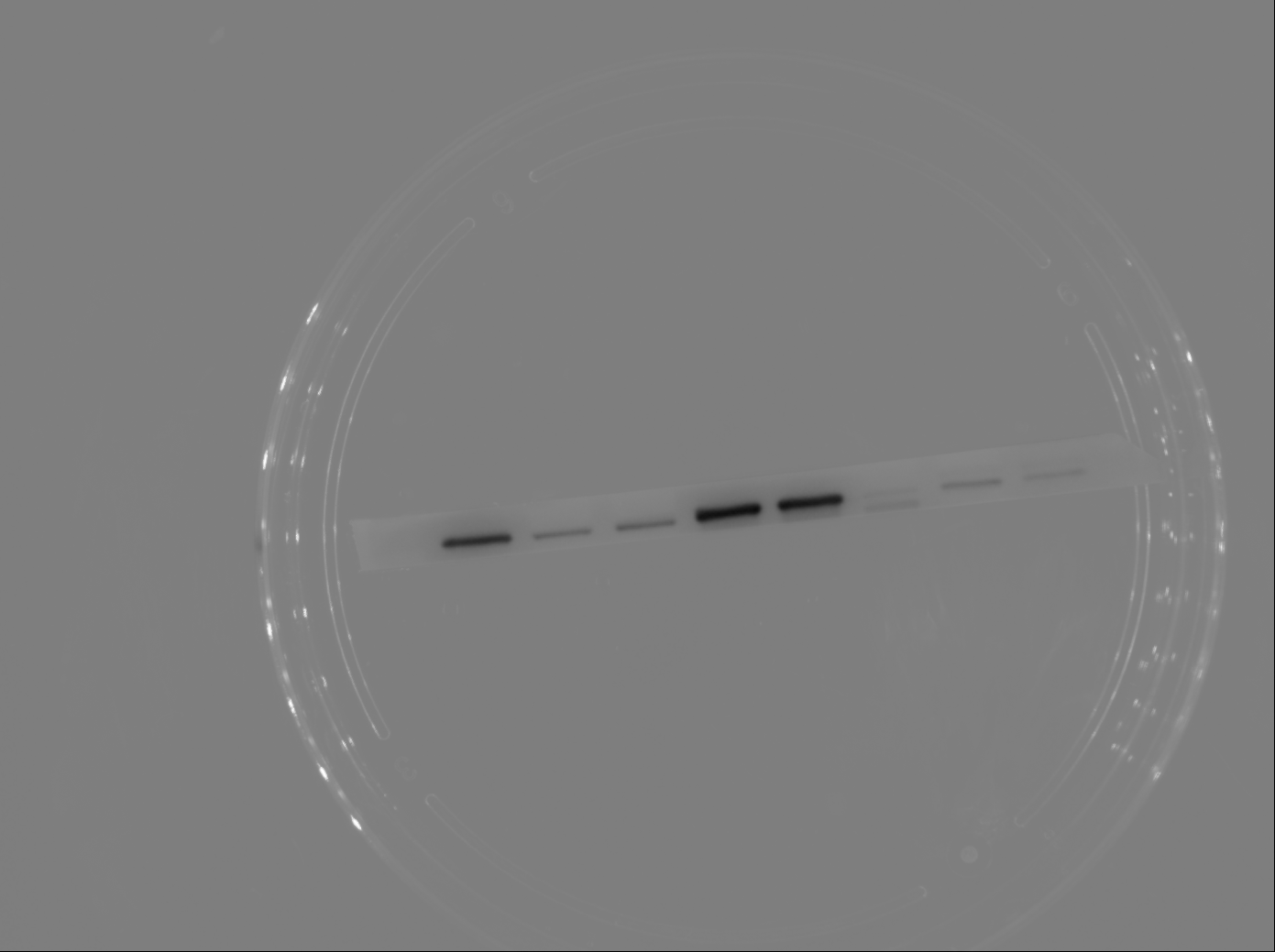


The expression level of AKAP7 protein in T24 cells was detected by WB after knockdown

T24NC sh1 sh2 sh3 NC sh1 sh2 sh3


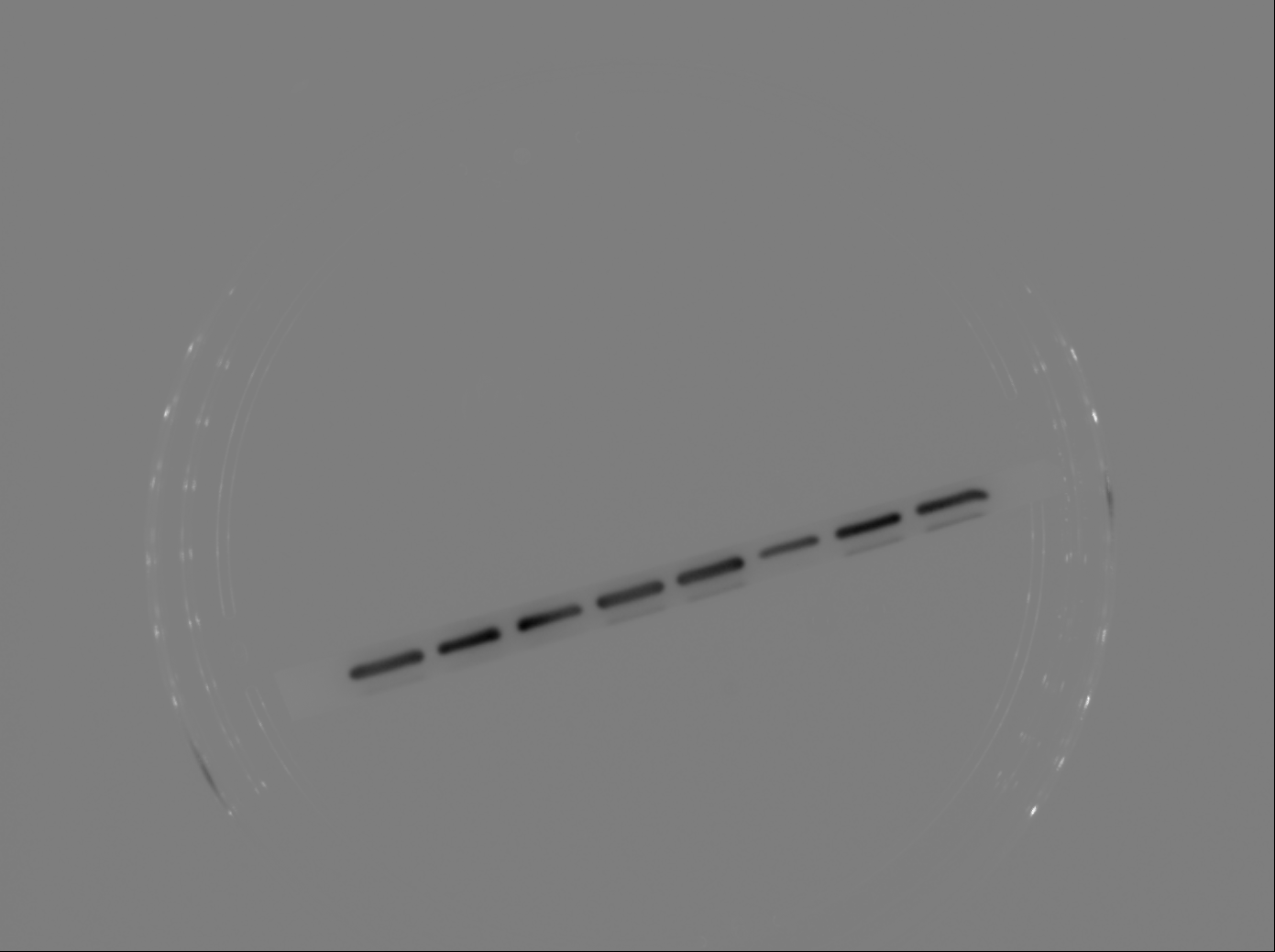


The expression level of GAPDH protein in T24 cells was detected by WB after knockdown
