# Supplementary material for: Uncovering the potential functions of lymph node metastasis-associated aberrant methylation differentially expressed genes and their association with the immune infiltration and prognosis in bladder urothelial carcinoma
Source: PeerJ. 2023 Apr 24;11:e15284. doi: 10.7717/peerj.15284 (PMC10135411; doi:10.7717/peerj.15284)
Supplement: Supplemental Information 7 [file peerj-11-15284-s007.docx]

**Bioinformatics data is downloaded from the open public database. The website of all downloaded data is as follows:**

**1. TCGA-BLCA-** **gene expression RNAseq (FPKM)**

https://xenabrowser.net/datapages/?dataset=TCGA-BLCA.htseq_fpkm.tsv&host=https%3A%2F%2Fgdc.xenahubs.net&removeHub=https%3A%2F%2Fxena.treehouse.gi.ucsc.edu%3A443

**2. TCGA-BLCA- Illumina Human Methylation 450**

https://xenabrowser.net/datapages/?dataset=TCGA-BLCA.methylation450.tsv&host=https%3A%2F%2Fgdc.xenahubs.net&removeHub=https%3A%2F%2Fxena.treehouse.gi.ucsc.edu%3A443

**3. TCGA-BLCA-copy number (gene-level)**

https://xenabrowser.net/datapages/?dataset=TCGA-BLCA.gistic.tsv&host=https%3A%2F%2Fgdc.xenahubs.net&removeHub=https%3A%2F%2Fxena.treehouse.gi.ucsc.edu%3A443

**4. TCGA-BLCA-somatic mutation (SNPs and small INDELs)**

https://xenabrowser.net/datapages/?dataset=TCGA-BLCA.varscan2_snv.tsv&host=https%3A%2F%2Fgdc.xenahubs.net&removeHub=https%3A%2F%2Fxena.treehouse.gi.ucsc.edu%3A443

**5. GEO-** **GSE13507**

https://ftp.ncbi.nlm.nih.gov/geo/series/GSE13nnn/GSE13507/matrix/
